# Antibiotic Consumption During the Coronavirus Disease 2019 Pandemic and Emergence of Carbapenemase-Producing *Klebsiella pneumoniae* Lineages Among Inpatients in a Chilean Hospital: A Time-Series Study and Phylogenomic Analysis

**DOI:** 10.1093/cid/ciad151

**Published:** 2023-07-05

**Authors:** Kasim Allel, Anne Peters, José Conejeros, José R W Martínez, Maria Spencer-Sandino, Roberto Riquelme-Neira, Lina Rivas, Pamela Rojas, Cristian Orellana Chea, Patricia García, Rafael Araos, Olivia McGovern, Twisha S Patel, Cesar A Arias, Fernanda C Lessa, Eduardo A Undurraga, José M Munita

**Affiliations:** Department of Disease Control, London School of Hygiene and Tropical Medicine, London, United Kingdom; Multidisciplinary Initiative for Collaborative Research On Bacterial Resistance (MICROB-R), Santiago, Chile; Institute for Global Health, University College London, London, United Kingdom; Multidisciplinary Initiative for Collaborative Research On Bacterial Resistance (MICROB-R), Santiago, Chile; Genomics and Resistant Microbes (GeRM), Facultad de Medicina Clínica Alemana, Instituto de Ciencias e Innovación en Medicina (ICIM), Universidad del Desarrollo, Santiago, Chile; Multidisciplinary Initiative for Collaborative Research On Bacterial Resistance (MICROB-R), Santiago, Chile; Multidisciplinary Initiative for Collaborative Research On Bacterial Resistance (MICROB-R), Santiago, Chile; Genomics and Resistant Microbes (GeRM), Facultad de Medicina Clínica Alemana, Instituto de Ciencias e Innovación en Medicina (ICIM), Universidad del Desarrollo, Santiago, Chile; Multidisciplinary Initiative for Collaborative Research On Bacterial Resistance (MICROB-R), Santiago, Chile; Genomics and Resistant Microbes (GeRM), Facultad de Medicina Clínica Alemana, Instituto de Ciencias e Innovación en Medicina (ICIM), Universidad del Desarrollo, Santiago, Chile; Multidisciplinary Initiative for Collaborative Research On Bacterial Resistance (MICROB-R), Santiago, Chile; Genomics and Resistant Microbes (GeRM), Facultad de Medicina Clínica Alemana, Instituto de Ciencias e Innovación en Medicina (ICIM), Universidad del Desarrollo, Santiago, Chile; Núcleo de Investigaciones Aplicadas en Ciencias Veterinarias y Agronómicas, Facultad de Medicina Veterinaria y Agronomía, Universidad de las Américas, Santiago, Chile; Multidisciplinary Initiative for Collaborative Research On Bacterial Resistance (MICROB-R), Santiago, Chile; Genomics and Resistant Microbes (GeRM), Facultad de Medicina Clínica Alemana, Instituto de Ciencias e Innovación en Medicina (ICIM), Universidad del Desarrollo, Santiago, Chile; Hospital Padre Hurtado, Santiago, Chile; Hospital Padre Hurtado, Santiago, Chile; Multidisciplinary Initiative for Collaborative Research On Bacterial Resistance (MICROB-R), Santiago, Chile; Departamento de Laboratorios Clínicos, Escuela de Medicina, Universidad Católica de Chile, Santiago, Chile; Multidisciplinary Initiative for Collaborative Research On Bacterial Resistance (MICROB-R), Santiago, Chile; Genomics and Resistant Microbes (GeRM), Facultad de Medicina Clínica Alemana, Instituto de Ciencias e Innovación en Medicina (ICIM), Universidad del Desarrollo, Santiago, Chile; Division of Healthcare Quality Promotion, Centers for Disease Control and Prevention, Atlanta, Georgia, USA; Division of Healthcare Quality Promotion, Centers for Disease Control and Prevention, Atlanta, Georgia, USA; Division of Infectious Diseases, Houston Methodist Hospital, Texas, USA; Division of Healthcare Quality Promotion, Centers for Disease Control and Prevention, Atlanta, Georgia, USA; Multidisciplinary Initiative for Collaborative Research On Bacterial Resistance (MICROB-R), Santiago, Chile; Escuela de Gobierno, Pontificia Universidad Católica de Chile, Santiago, Chile; Centro de Investigación para la Gestión Integrada del Riesgo de Desastres (CIGIDEN), Chile; Canadian Institute for Advanced Research (CIFAR) Azrieli Global Scholars Program, CIFAR, Toronto, Canada; Multidisciplinary Initiative for Collaborative Research On Bacterial Resistance (MICROB-R), Santiago, Chile; Genomics and Resistant Microbes (GeRM), Facultad de Medicina Clínica Alemana, Instituto de Ciencias e Innovación en Medicina (ICIM), Universidad del Desarrollo, Santiago, Chile; Hospital Padre Hurtado, Santiago, Chile

**Keywords:** Antimicrobial resistance, Antibiotic consumption, COVID-19, Carbapenemase-producing organisms, *Klebsiella pneumoniae*

## Abstract

**Background:**

The impact of coronavirus disease 2019 (COVID-19) on antimicrobial use (AU) and resistance has not been well evaluated in South America. These data are critical to inform national policies and clinical care.

**Methods:**

At a tertiary hospital in Santiago, Chile, between 2018 and 2022, subdivided into pre- (3/2018–2/2020) and post–COVID-19 onset (3/2020–2/2022), we evaluated intravenous AU and frequency of carbapenem-resistant Enterobacterales (CRE). We grouped monthly AU (defined daily doses [DDD]/1000 patient-days) into broad-spectrum β-lactams, carbapenems, and colistin and used interrupted time-series analysis to compare AU during pre- and post-pandemic onset. We studied the frequency of carbapenemase-producing (CP) CRE and performed whole-genome sequencing analyses of all carbapenem-resistant (CR) *Klebsiella pneumoniae* (CR*Kpn*) isolates collected during the study period.

**Results:**

Compared with pre-pandemic, AU (DDD/1000 patient-days) significantly increased after the pandemic onset, from 78.1 to 142.5 (*P* < .001), 50.9 to 110.1 (*P* < .001), and 4.1 to 13.3 (*P* < .001) for broad-spectrum β-lactams, carbapenems, and colistin, respectively. The frequency of CP-CRE increased from 12.8% pre–COVID-19 to 51.9% after pandemic onset (*P <* .001). The most frequent CRE species in both periods was CR*Kpn* (79.5% and 76.5%, respectively). The expansion of CP-CRE harboring *bla*_NDM_ was particularly noticeable, increasing from 40% (n = 4/10) before to 73.6% (n = 39/53) after pandemic onset (*P <* .001). Our phylogenomic analyses revealed the emergence of two distinct genomic lineages of CP-CR*Kpn*: ST45, harboring *bla*_NDM_, and ST1161, which carried *bla*_KPC_.

**Conclusions:**

AU and the frequency of CP-CRE increased after COVID-19 onset. The increase in CP-CR*Kpn* was driven by the emergence of novel genomic lineages. Our observations highlight the need to strengthen infection prevention and control and antimicrobial stewardship efforts.

## Teaser

The increased usage of carbapenems and broad-spectrum β-lactams during the COVID-19 pandemic was associated with a higher prevalence of carbapenemase-producing *Klebsiella pneumoniae* in a public hospital in Chile. We observed emergence and spread of *bla_NDM_* ST45 during the pandemic.

Antimicrobial resistance (AMR) constitutes a major health crisis causing substantial global disease and economic burden worldwide [1–3]. A recent report estimated 1.2 million deaths directly attributable to AMR in the year immediately prior to the emergence of coronavirus disease 2019 (COVID-19) [1]. Further, the impact of AMR is expected to increase, with estimates of approximately 10 million global annual AMR-related deaths by 2050 [4]. The World Health Organization (WHO) declared AMR as one of the most critical public health threats of the century [5].

COVID-19 led to a sharp increase in hospitalizations, a large proportion of which corresponded to high-complexity patients requiring admission to intensive care units (ICUs), invasive procedures, and prolonged hospital stays, in addition to shortages of healthcare personnel and protective equipment, especially early in the pandemic [6–8]. There is growing concern that COVID-19 might have resulted in higher antimicrobial use (AU) and in lapses in infection prevention and control (IPC) practices, both of which could have accelerated the spread of AMR [9–12]. Recent studies showed an escalation in AU during the pandemic, with up to 74.6% of patients with COVID-19 receiving one or more antibiotics [13, 14], despite the relatively low occurrence of secondary bacterial coinfections [15, 16]. The most frequently prescribed antibiotics were β-lactams (30%), fluoroquinolones (20%), and macrolides (18.9%) [13]. One study reported a significant increase in the use of broad-spectrum β-lactams (eg, cefepime, piperacillin/tazobactam, and carbapenems) and other last-resort antibiotics (eg, colistin and ceftazidime/avibactam) during the first pandemic peak [17].

Carbapenem-resistant Enterobacterales (CRE) are listed as critical-priority pathogens by the WHO [18]. A report from the US Centers for Disease Control and Prevention highlighted increases in both hospital-onset infections due to CRE and AU in inpatient settings during the first year of the pandemic [19]. Carbapenemase-producing (CP) CRE (CP-CRE) are particularly concerning as they harbor highly efficient enzymes often contained on mobile genetic elements that facilitate their spread, posing a daunting challenge for clinicians and IPC teams. A recent report alerted about an increased detection of CP-CRE after the COVID-19 pandemic in Latin America [20]. However, the magnitude of the impact of COVID-19 in the emergence of AMR remains unknown.

In Chile, official reports have shown that the most important CRE is carbapenem-resistant (CR) *Klebsiella pneumoniae (*CR*Kpn*), with a prevalence of approximately 35–40%. However, in contrast to other Latin American countries, the prevalence of CP-CRE prior to the pandemic was conspicuously low in Chile [21]. In this study, we evaluated the potential impact of the COVID-19 pandemic on AU and CRE. Moreover, we assessed the emergence of CP-CRE following the COVID-19 pandemic onset.

## METHODS

### Study Design and Sample Analysis

We collected hospital-wide data on AU and the frequency of CRE isolation in a public tertiary-care hospital in Santiago, Chile, with 391 beds and a catchment area of approximately 423 000 population (annual hospital discharges: ∼24 300) from March 2018 until March 2022. For context, the first patient with COVID-19 in Chile was diagnosed on 3 March 2020, and antimicrobial stewardship and IPC practices remained unchanged during the pandemic. We compared two years before the pandemic (pre–COVID-19, March 2018–February 2020) with two years after the onset of COVID-19 in Chile (COVID-19, March 2020–February 2022), combining various datasets and analytical strategies.

### Data Collection and Processing

Data were abstracted from the hospital's epidemiological and pharmacy records and included total number of beds, patient discharges, patient-days, and intravenous AU for all adult patients admitted to acute care wards during the study period. Acute care wards refer to any patient admitted from the emergency department or by a general practitioner, along with those electively admitted for a surgical procedure. Additionally, we obtained data on monthly ICU admissions and laboratory-confirmed COVID-19 discharges of adult subjects. Antimicrobial use was expressed in defined daily doses (DDDs) per 1000 patient-days and calculated for each intravenous compound as per WHO recommendations [22]. Antibiotics were classified into three groups: (1) broad-spectrum β-lactams (ie, ceftazidime, cefepime, piperacillin/tazobactam, ertapenem, meropenem, imipenem), (2) carbapenems (ie, imipenem, meropenem, and ertapenem), and (3) colistin, a drug frequently used against CP-CRE. Antibiotics evaluated in the study are presented individually in Supplementary Figure 1.

Throughout the study period, we prospectively collected all clinical CRE isolates (ie, nonsusceptible to ≥1 carbapenem as per Clinical and Laboratory Standards Institute [CLSI] 2022) recovered from invasive infections (ie, bloodstream, sterile fluids, or tissues). Isolates were sent to a central laboratory where species identification was reconfirmed by MALDI-TOF (matrix-assisted laser desorption/ionization–time of flight) mass spectrometry. The antibiotic susceptibility profile was reconfirmed using the disk diffusion method following CLSI 2022 [23]. Testing was performed using a multiplex polymerase chain reaction (PCR) designed to detect the three carbapenemases most frequently reported in the country (ie, *Klebsiella pneumoniae* carbapenemase [*bla*_KPC_], New Delhi metallo-β-lactamase [*bla*_NDM_], and Verona integron–encoded metallo-β-lactamase [*bla*_VIM_]) and was performed in all CRE isolates. Finally, given their high frequency and clinical relevance, we performed whole-genome sequencing (WGS) on all CR*Kpn* isolates recovered during the study period.

### Statistical Analyses

Descriptive statistics were used to visualize monthly AU, ICU admissions, and COVID-19 patient discharges. A second-order polynomial fit was adjusted to the data as it presented the best goodness-of-fit (according to the Akaike information criterion [AIC]). The AU rate for each antibiotic group expressed by DDDs per 1000 patient-days was compared between pre- and post-pandemic onset. To further understand AU over time, we calculated a baseline average monthly AU between March 2018 and February 2019. Using this information, we estimated the monthly percentage change for March 2019–February 2020 (pre-pandemic) and for the two years post-pandemic onset (March 2020–February 2022).

We used interrupted time-series analyses for each antibiotic group [24, 25] to evaluate the impact of COVID-19 on AU, adjusting for seasonality and autocorrelation. First, we logarithmically transformed AU rates to adjust their variance over time and computed a first-order differentiation between consecutive time points to correct stationarity. Subsequently, we tested autocorrelation and seasonality among AU group variables [25]. We used an autoregressive integrated moving average (ARIMA) approach through an automated algorithm, based on the best goodness-of-fit reported (eg, lowest AIC/Bayesian information criterion [BIC]), resulting in a seasonal ARIMA (1,0,0) (0,1,1) model [24]. A seasonal ARIMA model is classified as an ARIMA(p,d,q) x (P,D,Q), where (p,d,q) refers to the seasonal and (P,D,Q) to the non-seasonal component. P or p = number of seasonal autoregressive terms, D or d = number of seasonal differences, Q or q = number of seasonal moving average terms. The interrupted side of the model comprised step change and ramp components, derived from any random shift and slope changes in AU over time after the pandemic onset [25]. Finally, we generated a counterfactual scenario related to a hypothetical absence of the COVID-19 pandemic to contrast observed and estimated AU through the backward prediction of the time series as if no random shift and slope changes would have existed. Analyses were conducted using R version 3.2.1 (R Foundation for Statistical Computing).

### Whole-Genome Sequencing and Phylogenomic Analyses

We performed WGS using Illumina MiSeq with the Illumina DNA library prep kit (Illumina, Inc). We used FASTQC and MultiQC to determine the read's quality, and Trimmomatic to pair the reads [26, 27]. The genomes were assembled de novo with SPAdes, and the quality of the assemblies was assessed with QUAST [28, 29]. We used MLST 2.19.0 [30] and ABRicate v1.0.1 15 to determine the sequence type (ST) and the presence of carbapenemases. We annotated genome assemblies with Bakta [31] and evaluated the pangenome using Roary v3.13.0 [32]. A maximum likelihood phylogenomic tree was performed using a core genome definition of 99% with RAxML 8.2.12 [33]. Finally, a recombination-free phylogenomic tree was generated with Clonal Frame ML v1.12 [34] and visualized with the interactive Tree Of Life (iTOL) tool [35].

### Ethics

Our study was approved by the Research Ethics Committee of the Clinica Alemana, Universidad del Desarrollo Faculty of Medicine (Institutional Review Board [IRB] 2021-24, Protocol number #UIEC1047).

## RESULTS

### Hospital Characteristics and Epidemiological Analyses

The first patient with COVID-19 in Chile was diagnosed in early March 2020 and the first pandemic wave peaked in June 2020 [36]. During this peak, our hospital discharged 530 patients with COVID-19 (Supplementary Figure 2*A*). The total number of beds and average monthly hospital discharges did not significantly vary during the study period. ICU admissions substantially increased after the pandemic onset, with an average of 11 and 25 ICU admissions in the pre- and post-pandemic period, respectively (*P* < .001). Most ICU admissions (80%) during the pandemic period were patients aged older than 60 years (Supplementary Figure 2*B*).

### Antibiotic Use Over Time and Impact of COVID-19

**Figure 1.**
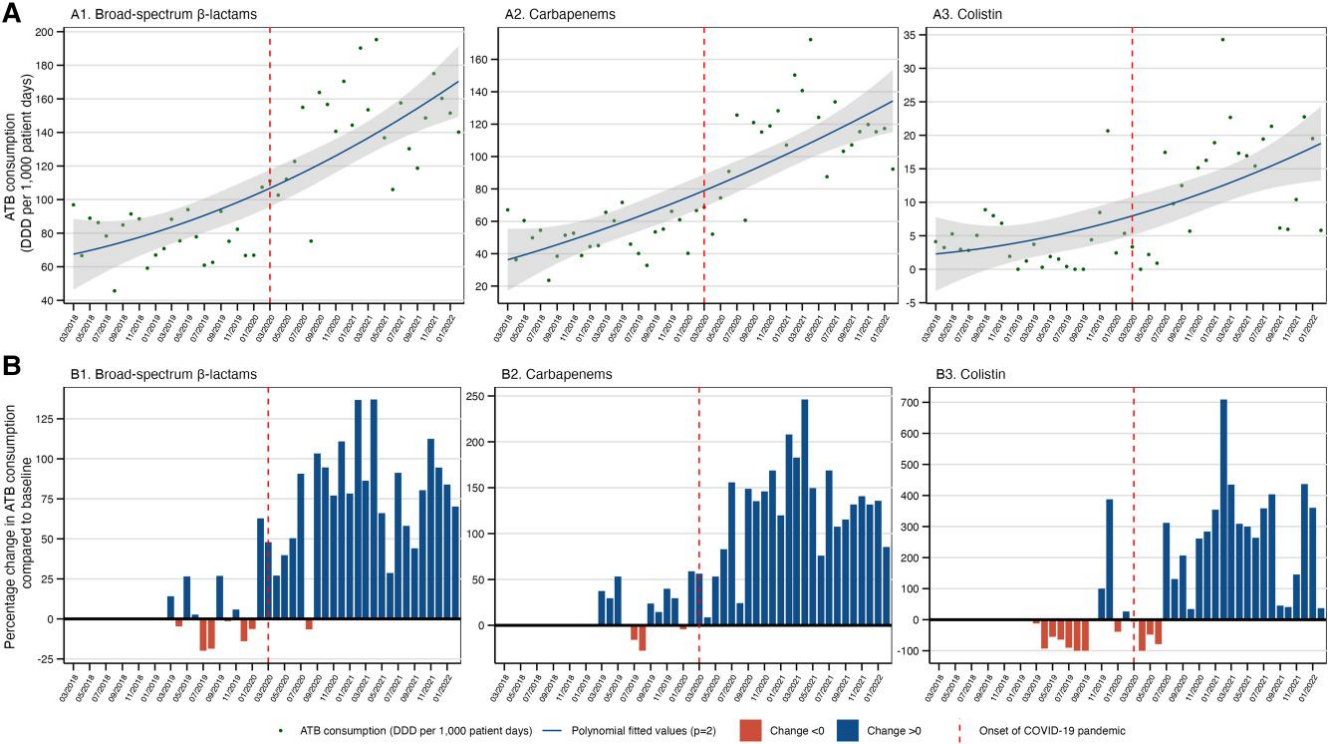
Hospital-wide antibiotic consumption in DDD per 1000 patient-days (*A*) and monthly percentage change over time (*B*), by antibiotics group, 2018–2022. Colistin is classified as a compound active against CP organisms. Broad-spectrum β-lactam ATBs include piperacillin/tazobactam, cefepime, ceftazidime, meropenem, and imipenem. Carbapenems include imipenem, meropenem, and ertapenem. (*B*) Percentage change in antibiotic consumption over time (compared with the average antibiotic consumption between March 2018 and February 2019). Abbreviations: ATB, antibiotic; COVID-19, coronavirus disease 2019; CP, carbapenemase-producing; DDD, defined daily dose.

Compared with pre–COVID-19, we observed a significant increase in mean DDDs per 1000 patient-days during COVID-19, with an overall higher AU of broad-spectrum β-lactams (78.1 vs 142.5; *P* < .001), carbapenems (50.9 vs 110.1; *P* < .001), and colistin (4.1 vs 13.3; *P* < .001) (Figure 1 and Table 1). Noticeably, the highest surge in AU of broad-spectrum β-lactams, carbapenems, and colistin was observed approximately 12 months after the pandemic onset, peaking at 137%, 246%, and 705%, respectively (Figure 1*B* ). The monthly variation in AU for individual antibiotics is provided in Supplementary Figures 1 and 3. Cefepime, ertapenem, imipenem, meropenem, and colistin drove the increasing trend in consumption among the different antibiotic groups (Supplementary Figure 3). The AU of colistin, imipenem, and meropenem increased after COVID-19's onset in the ICU and in general wards, but remained stable in the emergency department (Supplementary Figure 4).

**Table 1.** Average Antibiotic Use Before (March 2018–February 2020) and After COVID-19's Onset (March 2020–February 2022) by Group

Antibiotic Group and Pandemic Onset	Mean	SD	Min	P25	P50	P75	Max	Percentage Variation in Mean Antibiotic Consumption
Broad-spectrum β-lactams								
Before	78.13	14.52	45.64	66.82	78.08	88.60	107.41	…
After	142.45	28.74	75.29	121.72	146.47	158.25	195.29	82.3%
Carbapenems								
Before	50.90	12.57	23.56	40.21	52.05	60.58	71.65	…
After	110.07	28.14	52.07	91.89	115.29	124.53	172.20	116.2%
Colistin								
Before	4.15	4.44	0.00	1.45	3.10	5.30	20.66	…
After	13.34	8.46	0.00	5.93	15.28	19.03	34.29	221.5%

Data are presented as percentages. Times defined as before and after COVID-19's onset are equally sized (12 months after and before COVID-19).

Abbreviations: COVID-19, coronavirus disease 2019; Max, maximum; Min, minimum; P25, P50, and P75, 25th, 50th, and 75th percentiles, respectively; SD, standard deviation.

There was an immediate increase in AU of broad-spectrum β-lactams and carbapenems after the pandemic onset (step change coefficient [coeff] = .38; 95% confidence interval [CI] = .17–.59; *P* < .001; and step change coeff = .49; 95% CI = .09–.88; *P* = .016, respectively). We observed a significant shift in the slope of broad-spectrum β-lactam usage over time (slope change coeff =0.03; 95% CI = .01–.06; *P* = .006) (Table 2). In contrast, use of carbapenems and colistin was mainly related to AU in previous months (autoregressive order [AR] coeff), and no significant association was observed for the slope change (coeff = .03; 95% CI = −.02 to .08; *P* = .17; and coeff = .04; 95% CI = −.14 to .23; *P* = .65, respectively). A comparison between the observed AU and the theoretical expected values (counterfactual) in the absence of COVID-19 is presented in Supplementary Figure 5. After the first year of COVID-19, our analyses revealed an estimated excess (ie, difference between observed and estimated counterfactual) of 77, 73, and 18 DDDs per 1000 patient-days for broad-spectrum β-lactams, carbapenems, and colistin, respectively.

**Table 2.** Interrupted Time-Series Model Results for Antibiotic Consumption Using a Seasonal ARIMA (1,0,0)(0,1,1) Approach

Term	Coeff.	SE	95% CI	*P*
(A) Broad-spectrum β-lactams				
AR (1)	−.08	.17	−.42, .25	.999
SM (1)	−.73	.57	−1.84, .38	.999
Onset of COVID-19 pandemic				
Step change	.38	.11	.17, .59	<.001
Ramp (slope change)	.03	.01	.01, .06	.006
Sigma^2^ = 0.04363: log likelihood = 3.33, AIC = 3.33, BIC = 11.25				
(B) Carbapenems				
AR (1)	.37	.18	.02, .72	.037
SM (1)	−.99	.62	−2.21, .21	.999
Onset of COVID-19 pandemic				
Step change	.49	.20	.09, .88	.016
Ramp (slope change)	.03	.03	−.02, .08	.175
Sigma^2^ = 0.06279: log likelihood = −7.52, AIC = 25.03, BIC = 32.95				
(C) Colistin				
AR (1)	.38	.16	.06, .71	.019
SM (1)	−.99	.39	−1.77, -.23	.428
Onset of COVID-19 pandemic				
Step change	.73	.75	−.74, 2.19	.336
Ramp (slope change)	.04	.09	−.14, .23	.653
Sigma^2^ = 0.7965: log likelihood = −53.26, AIC = 116.52, BIC = 124.43				

The AR term refers to autoregressive order; the Ramp coefficient indicates the increment at each time point of the time series after the COVID-19 pandemic. The Step change coefficient indicates the augment rate immediately following the intervention; SM is for seasonal moving average. The model used the logged form of the difference in antibiotic consumption over time (by group); hence, coefficients should be transformed for interpretation. The logged time series and autocorrelation functions were computed to indicate if the time series was stationary. Our analysis of the model’s residuals indicated they were uncorrelated and had a zero mean. Significance level, α = 5.

Abbreviations: AIC, Akaike information criterion; ARIMA, autoregressive integrated moving average; BIC, Bayesian information criterion; CI, confidence interval; Coeff., coefficient; COVID-19, coronavirus disease 2019; SE, standard error.

### Microbiological and Molecular Characterization of the CRE and CR*Kpn* Collection

**Figure 2.**
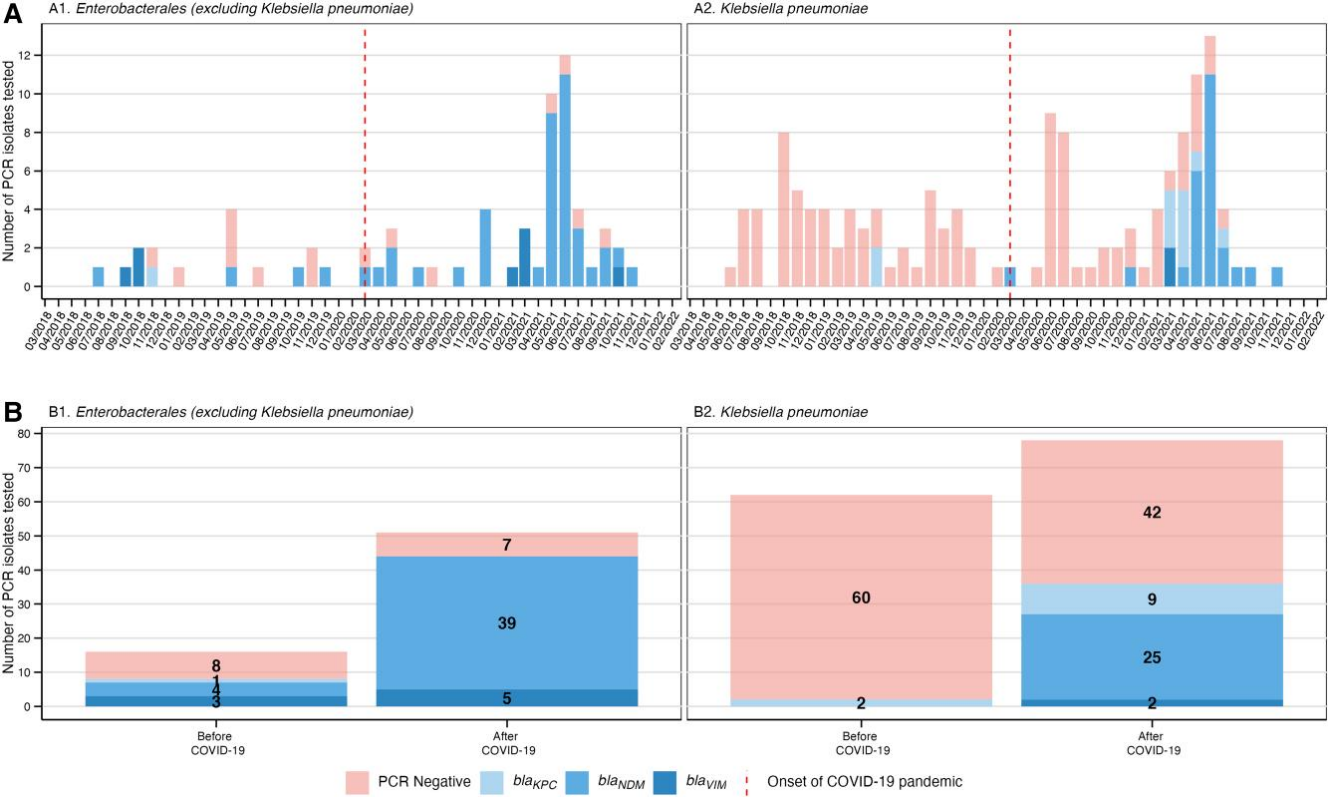
(*A*, *B*) Carbapenem-resistant Enterobacterales and carbapenem-resistant *Klebsiella pneumoniae* over the study period and proportion of isolates harboring *bla*_KPC_, *bla*_NDM_, or *bla*_VIM_ carbapenemases before and after the COVID-19 pandemic, 2018–2022. Abbreviations: *bla*_KPC_, *Klebsiella pneumoniae* carbapenemase; *bla*_NDM_, New Delhi metallo-β-lactamase; *bla*_VIM_, Verona integron-encoded metallo-β-lactamase; COVID-19, coronavirus disease 2019; PCR, polymerase chain reaction.

Overall, 180 CRE isolates from 139 patients were collected during the study period: 78 (43%) pre–COVID-19 and 102 (57%) during COVID-19. The number of isolates per patient did not change significantly before and during COVID-19 (1.37 and 1.24, respectively). The majority of CRE recovered in both periods corresponded to CR*Kpn* (62/78 [79.5%] and 78/102 [76.5%], respectively), followed by CR-*Enterobacter cloacae* complex (16/78 [20.5%] and 16/102 [15.7%], respectively) (Figure 2 and Supplementary Table 1). The proportion of CRE found to harbor *bla*_KPC_, *bla*_NDM_, or *bla*_VIM_ increased from 12.8% (10/78) pre–COVID-19 to 51.9% (53/102) during COVID-19 (*P* < .001) (Figure 2*B* ). Prior to the pandemic, only 2 CR*Kpn* isolates harbored any of these carbapenemases (ie, CP-CR*Kpn*), *bla_KPC_* specifically. The proportion of CP-CR*Kpn* isolates increased from 3.2% (2/62) pre–COVID-19 to 46.2% (36/78) during COVID-19 (*P* = .005) (Figure 2*B* ). Apart from CR*Kpn*, the proportion of other CRE isolates harboring a carbapenemase increased from 50% (n = 8/16) pre–COVID-19 to 70.8% (n = 17/24) during COVID-19 (Figure 2). A comparison of the antimicrobial susceptibility profiles between CP- and non–CP-CRE and CR*Kpn* is shown in Supplementary Figure 6. Notably, while 63 of 117 non–CP-CRE isolates were resistant to at least one of the three carbapenems (ertapenem: 96%, 112/117; meropenem: 58%, 68/117; imipenem: 8%, 9/117), most CP strains (99%, 62/63) exhibited resistance to all three carbapenems (Supplementary Figure 6).

Most organisms encoding *bla*_KPC_, *bla*_NDM_, or *bla*_VIM_, especially among CR*Kpn*, were recovered from March 2021 onward, precisely one year after the pandemic onset (Figure 2*A* ). The expansion of CP-CRE harboring *bla*_NDM_ was particularly noticeable, increasing from 40.0% (4/10) pre–COVID-19 to 73.6% (39/53) during COVID-19 (*P <* .001). Among CP-CR*Kpn, bla*_NDM_ increased from 0% to 69.4% (25/36), and among non–CP-CR*Kpn*, *bla*_NDM_ increased from 50.0% (4/8) to 82.4% (14/17), mostly driven by CR-*E. cloacae* (Figure 2 and Supplementary Table 1).

### Whole-Genome Sequencing and Phylogenomic Analyses of CR*Kpn*

**Figure 3.**
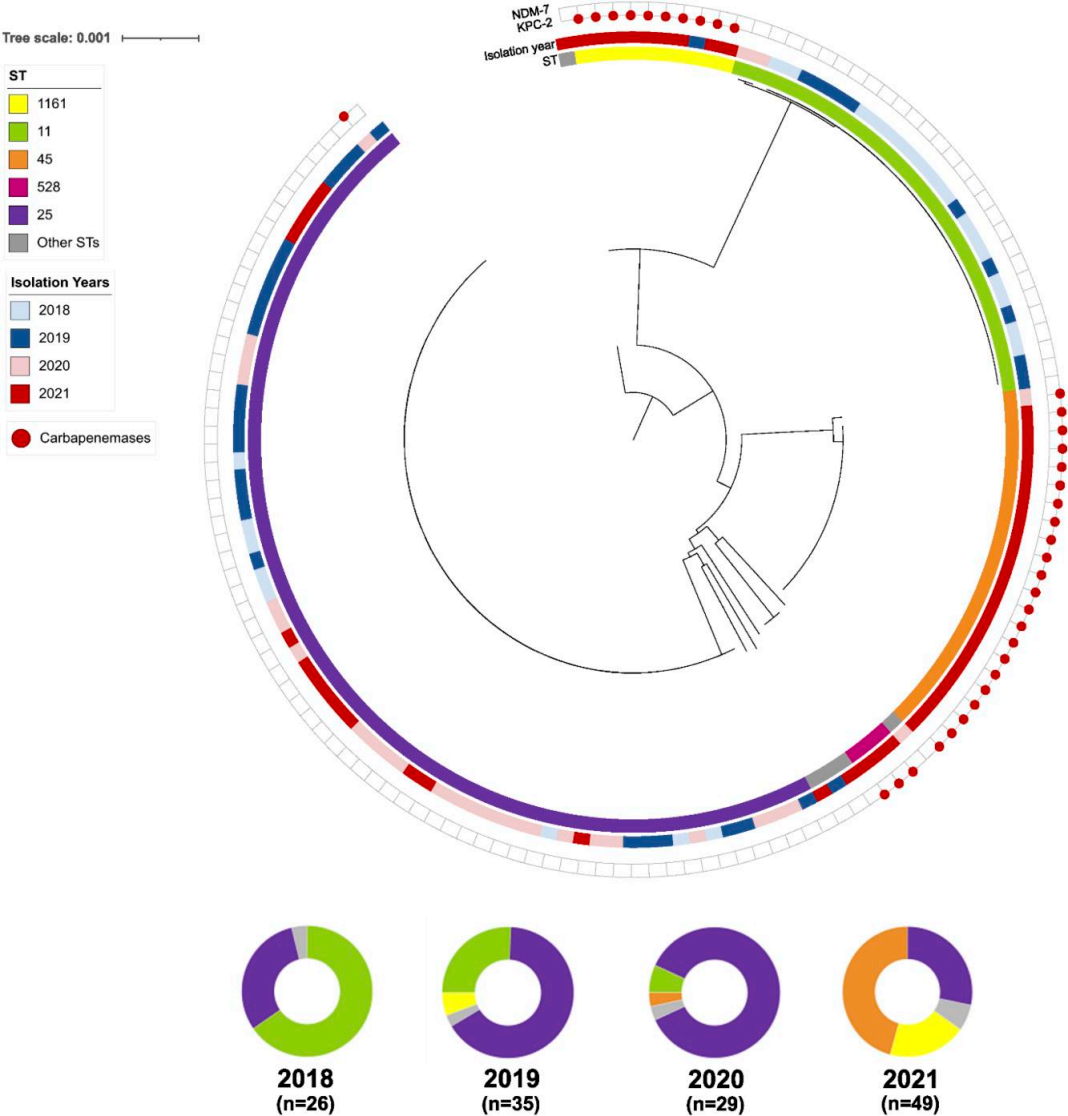
Genomic characterization of 140 carbapenem-resistant *Klebsiella pneumoniae* isolates. *Upper panel*: Maximum-likelihood recombination-free phylogenomic tree rooted to the midpoint of the genomic distances. The inner colored ring shows the ST; the external colored ring represents the year of isolation. The external red circles indicate the presence of the carbapenemase-encoding genes *bla*_KPC_, and *bla*_NDM_. *Lower panel*. Frequency ST by year of isolation. Abbreviations: *bla*_KPC_, *Klebsiella pneumoniae* carbapenemase; *bla*_NDM_, New Delhi metallo-β-lactamase; ST, sequence type.

Figure 3 shows a core genome-based phylogenomic reconstruction of 140 strains of CR*Kpn* recovered from the bloodstream, other sterile sites, or tissue from 2018 to 2021. Overall, the most frequently observed genomic lineages were ST25 (49.6%), ST11 (20.1%), ST45 (15.8%), and ST1161 (7.9%). After grouping genomes by year, our temporal analysis revealed critical clonal replacements over time (Figure 3). While ST11 accounted for the majority of isolates (69%) in 2018, we found a gradual replacement of this lineage for strains belonging to ST25 in 2019 and 2020. The ST25 lineage increased from 30.8% in 2018 to 68% and 92% in 2019 and 2020, respectively (Figure 3). After the first pandemic year, we observed the emergence of two lineages of CP-CR*Kpn*, ST45 and ST1161, which increased from 3.4% and 0% in 2020 to 42.9% and 18.4% in 2021 (Figure 3). Our genomic analyses revealed that all *bla*_KPC-2_-containing isolates belonged to the ST1161 lineage, as did the only carbapenemase-producing CR*Kpn* recovered before the COVID-19 pandemic (2019). On the other hand, while *bla*_NDM-7_ was predominantly found in CR*Kpn* isolates belonging to the ST45 lineage, this trait was also found in genomes belonging to other lineages such as ST528 (n = 3) and ST25 (n = 1). All *bla*_NDM-7_-producing CR*Kpn* organisms were recovered after the pandemic onset.

## DISCUSSION

Understanding the drivers of AMR is critical to prevent the spread of multidrug-resistant organisms. Our data from a large public hospital in Chile show an association of the COVID-19 pandemic with increases in broad-spectrum antibiotic use and CRE infections. Notably, during the pandemic period we observed a significant increase in the proportion of CP-CRE, which was particularly relevant for CP-CR*Kpn*, with an approximately 7-fold increase in isolates encoding *bla*_KPC_ or *bla*_NDM_. This increase was driven by the appearance of two distinct genomic lineages of CP-CR*Kpn*: ST1161 (harboring *bla*_KPC-2_) and ST45 (harboring *bla*_NDM-7_).

The increase observed in CP-CRE, and especially in *bla*_NDM_-harboring organisms, which was previously uncommon in Chile, has been reported in other Latin American countries during the pandemic [20]. In October 2021, the Pan American Health Organization issued an alert on the emergence of and increase in new combinations of carbapenemases in Enterobacterales in the region [37]. Although we did not find CRE harboring more than one carbapenemase, several countries in Latin America have reported the detection of dual-producers after the pandemic [20]. The rapid dissemination of CP-CR*Kpn* ST45 harboring *bla*_NDM-7_ observed in 2021 may suggest in-hospital transmission rather than multiple introductions. Hospitals, from different regions, reported challenges maintaining IPC practices, contributing to increases in healthcare-associated infections [38, 39].

Importantly, as shown by our data and official reports, our study was performed in a setting of low CP-CRE prevalence pre–COVID-19 [21], which provides a perfect setting to assess the COVID-19 impact on the emergence of CP organisms. Our phylogenomic analyses of CR*Kpn* revealed that the increase in CP-CR*Kpn* during 2021 was primarily driven by the emergence of two genomic lineages. ST1161 carried *bla*_KPC-2_, a class A enzyme frequently observed in CR*Kpn* in different parts of the world. In contrast, strains of ST45 harbored *bla*_NDM-7_, a class B metallo-enzyme against which there are very few, if any, reliable therapeutic options. While *bla*_NDM-7_ was also found in CP-CR*Kpn* from other genomic lineages (ie, ST25 and ST528), *bla*_KPC-2_ was only observed in ST1161, suggesting that *bla*_NDM-7_ could be located in a mobile genetic element that facilitates its horizontal transmission into different genomic lineages and perhaps species. Moreover, the fact that *bla_NDM_* was observed in non–*K. pneumoniae* CRE prior to the pandemic and increased during the pandemic, mainly driven by *E. cloacae* complex, may hint towards horizontal transmission of this genetic trait. The study of genomic platforms with long-read sequencing analyses and transmission dynamics is part of our future research endeavors.

In addition to an increase in CP organisms, we observed an increase in AU after the pandemic onset. Our findings are consistent with previous reports from China suggesting that approximately 70% of patients with COVID-19 received antibiotic treatment during the early stages of the pandemic [40, 41]. We observed a prolonged and consistent increase in broad-spectrum β-lactams, carbapenems, and colistin after the first pandemic wave. Antimicrobial use peaked soon after the first year since the pandemic onset and coincided with the increase in CP-CRE. While there is a temporal correlation, our data do not allow us to establish causality. Therefore, the role of the increases in AU in selecting for CRE in general, and CP-CRE in our hospital, remains unclear. Several studies have demonstrated AU to be an independent risk factor for CRE colonization, including a meta-analysis focused on CR*Kpn* [42,43]. Further studies are needed to evaluate the appropriateness and drivers of AU in the hospital and its role in the emergence of CP-CRE.

Our study has several limitations. First, we only performed PCR detection for *bla*_KPC_, *bla*_NDM,_ and *bla*_VIM_; therefore, it is possible that we missed other relevant carbapenemases, leading to an underestimation of the number of CP-CRE isolates. Indeed, a recent communication reported the first detection of *bla*_OXA-48_ in CR*Kpn* and *Escherichia coli* in Chile during the pandemic [36]. However, we did perform WGS in all CR*Kpn* and no other carbapenemases were observed in these analyses. Second, while we analyzed the genomes of all CR*Kpn* (which were by far the most frequent bacterial species), our WGS data did not include other organisms (eg, CR-*E. cloacae* complex), limiting our ability to draw conclusions about relevant observations such as the expansion of *bla*_NDM_-harboring organisms. Third, our analyses are ecological by nature and preclude conclusions regarding any causal effect. Although AU is one of the main drivers of AMR [44], our data do not allow us to rule out the influence of confounding factors, therefore hampering our ability to establish direct causality between AU increase and the emergence of CP-CRE.

Despite these limitations, this is the first report examining the temporal association between COVID-19 and its impact on AU and AMR in Chile. It draws attention to the emergence of genomic lineages of CP-CRE that pose treatment challenges and emphasizes the need for improved antibiotic stewardship and enhanced IPC measures to prevent their spread within healthcare facilities. The use of genomic surveillance provides data to help understand whether there were multiple introductions of new strains or if there is an expansion of a single strain, which hints towards healthcare transmission. It is not known whether *bla*_KPC-2_ ST1161 or *bla*_NDM-7_ ST45 CR*Kpn* will spread rapidly within Chilean or South American hospitals, but increased vigilance will be warranted.

In summary, our analyses show that AU rate and AMR increased during COVID-19 surges in Chile. Additional studies are necessary to understand the specific ways in which the burden of the pandemic affected AU and AMR rates and whether the increases in AU observed in our data directly increased the risk of AMR among our population. Our findings also highlight the need to build capacity for IPC and antimicrobial stewardship programs. As we move into next phase of the COVID-19 pandemic and recovery, it will be critical to emphasize the need for strong IPC programs, one of the cornerstones of a resilient healthcare system.

### Lesson Learned

Strengthening our capabilities to ensure appropriate AU, rapid genome-based surveillance of emerging multidrug-resistant pathogens, and efficient IPC programs is crucial to tackle AMR in the future.

## Supplementary Data

Supplementary materials are available at *Clinical Infectious Diseases* online. Consisting of data provided by the authors to benefit the reader, the posted materials are not copyedited and are the sole responsibility of the authors, so questions or comments should be addressed to the corresponding author.

## Supplementary Material

ciad151_Supplementary_Data

## Data Availability

Data are self-contained throughout the manuscript, full dataset is available upon request.

## Notes

***Author Contributions.*** Study design and analytical methods: A. P., K. A., J. R. W. M., R. R.-N., E. A. U., J. M. M. Data analyses: A. P., K. A., J. C. Manuscript writing: A. P., K. A., J. R. W. M., E. A. U., J. M. M. Data collection: A. P., M.S-S., L. R. Data interpretation, critical manuscript review, edition, and final approval: all authors. All authors attest they meet the ICMJE criteria for authorship and have approved the final article.

***Financial support.*** This research was partially supported by the Agencia Nacional de Investigación y Desarrollo (ANID) Beca de Doctorado en el Extranjero Becas Chile, Chile (grant number 73200098; to K. A.); Agencia Nacional de Investigación y Desarrollo ANID/ Fondo de Financiamiento de Centros de Investigación en Áreas Prioritarias (FONDAP) Centro Nacional deInvestigación para la Gestión Integrada de Desastres Naturales (CIGIDEN) (grant number 1522A0005; to E. A. U.); Fondo Nacional de Desarrollo Científico y Tecnológico (FONDECYT; grant number 1211933; to P. G., E. A. U., J. M. M); FONDECYT (grant number 1211947; to J. M. M.); and Canadian Institute for Advanced Research (CIFAR), under the Humans and Microbiome Programme to E. A. U.

***Supplement sponsorship.*** This article appears as part of the supplement “The Evolving Challenges of Antibiotic Resistance in Low- and Middle-Income Countries: Priorities and Solutions,” sponsored by the U.S. Centers for Disease Control and Prevention, and Health Security Partners.
