# Supplementary material for: Antibiotic Consumption During the Coronavirus Disease 2019 Pandemic and Emergence of Carbapenemase-Producing Klebsiella pneumoniae Lineages Among Inpatients in a Chilean Hospital: A Time-Series Study and Phylogenomic Analysis
Source: Clin Infect Dis. 2023 Jul 5;77(Suppl 1):S20–8. doi: 10.1093/cid/ciad151 (PMC10321701; doi:10.1093/cid/ciad151)
Supplement: ciad151_Supplementary_Data [file ciad151_supplementary_data.docx]

**SUPPLEMENTARY MATERIAL**

**Antibiotic Consumption During the COVID-19 Pandemic and Emergence of Carbapenemase-Producing Klebsiella pneumoniae Lineages Among Inpatients in a Chilean Hospital: A Time Series Study and Phylogenomic Analysis**

Kasim Allel, Anne Peters, José Conejeros, José RW Martínez, Maria Spencer-Sandino, Roberto Riquelme-Neira, Lina Rivas, Pamela Rojas, Patricia García, Rafael Araos, Olivia McGovern, Twisha S. Patel, Cesar A. Arias, Fernanda C. Lessa, Eduardo A. Undurraga, José M. Munita


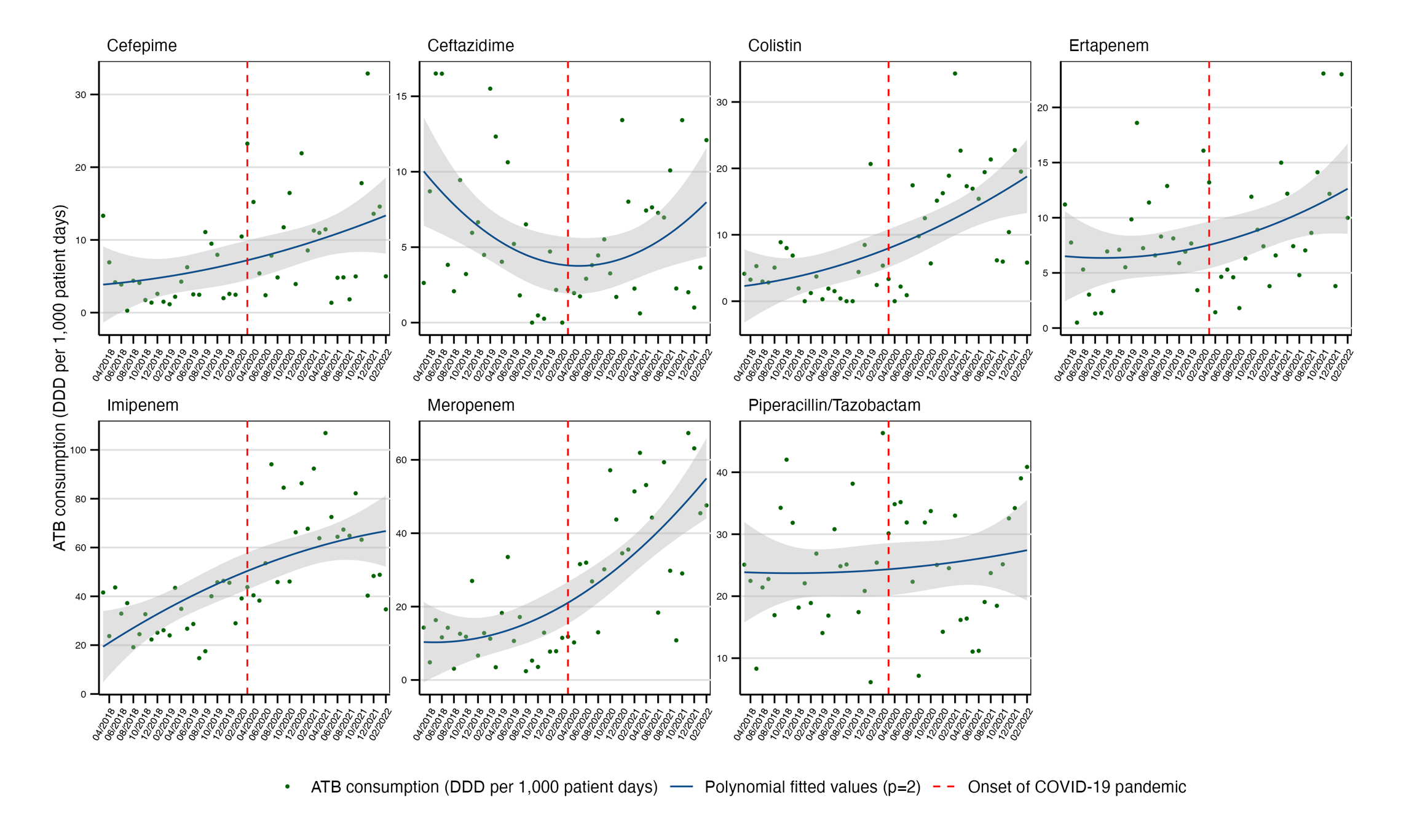
**Figure S1** Antibiotic consumption in DDDs per 1,000 patient-days over time for each antibiotic included in the study, 2018-2022

Notes: ATB= Antibiotic. DDD= Defined Daily Dose.

# **Figure S2.** Hospital discharge for COVID-19 and ICU admissions over the study period stratified by age group, 2018-2022


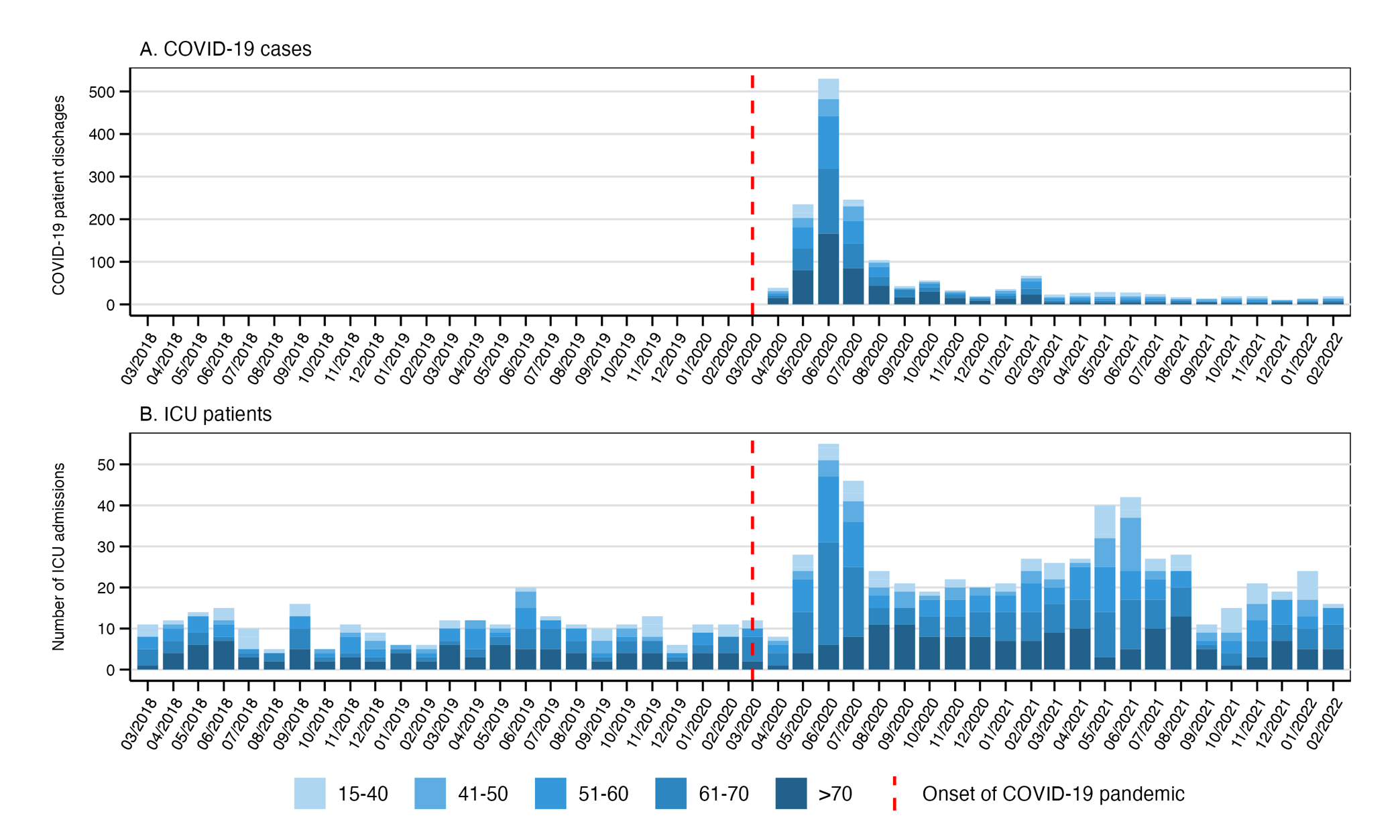


Notes: ICU, Intensive care unit; Boxes represent age groups and dashed red line the COVID-19 pandemic onset.


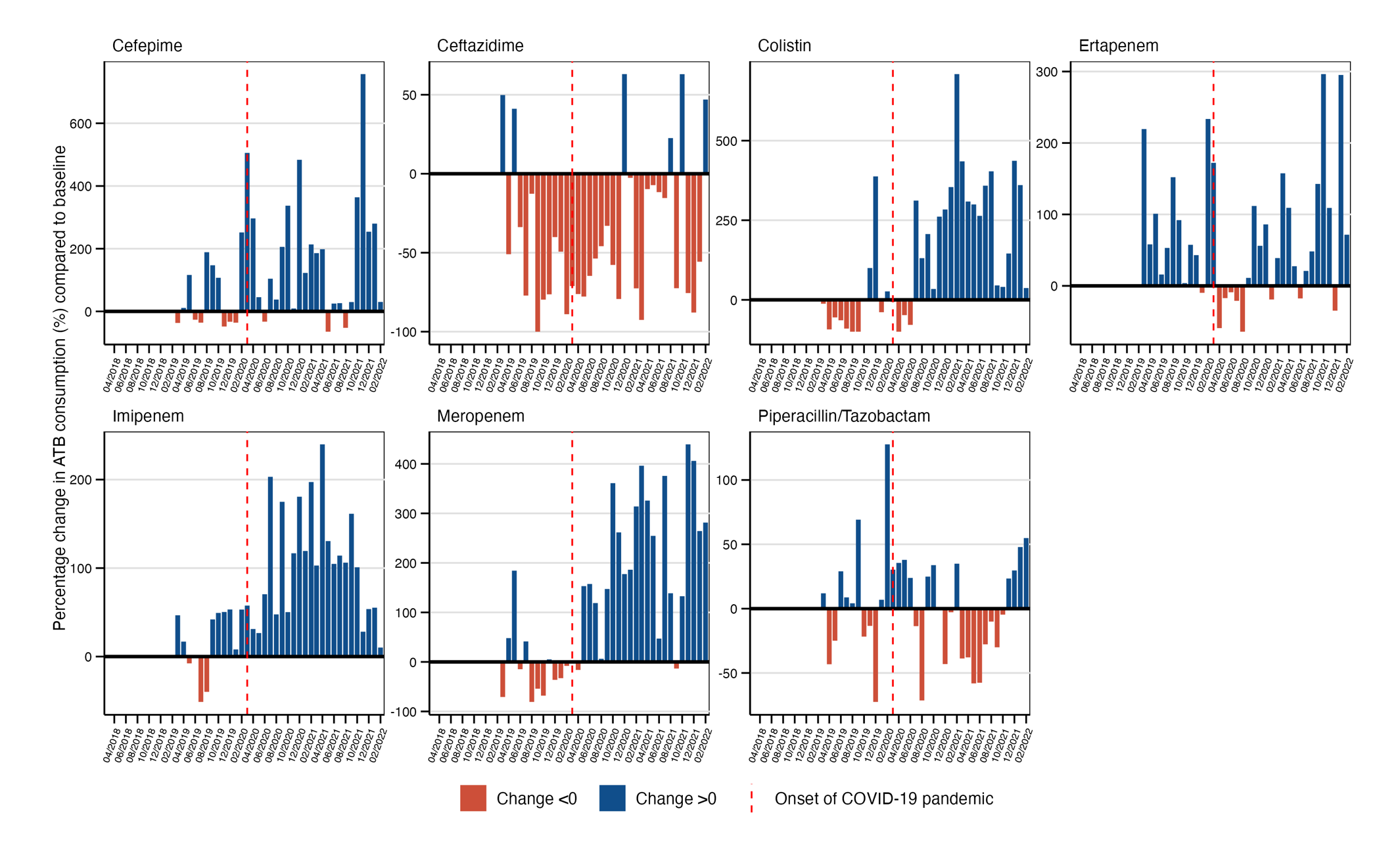
**Figure S3** Percentage change in antibiotic consumption over time (*compared to the average antibiotic consumption between March 2018 and February 2019*) by antibiotic

Notes: ATB= Antibiotic. Average antibiotic use between March 2018 and February 2019 was used as baseline.

**Figure S4** Antibiotic consumption in DDDs per 1,000 patient-days over time for each antibiotic included in the study and by type of ward, 2018-2022.

Notes: ATB= Antibiotic. DDD= Defined Daily Dose. ICU= Intensive Care Unit.

**Figure S5.** Counterfactual estimation of the time-series for each antibiotic consumption group under a hypothetical scenario of no COVID-19, 2018-2022


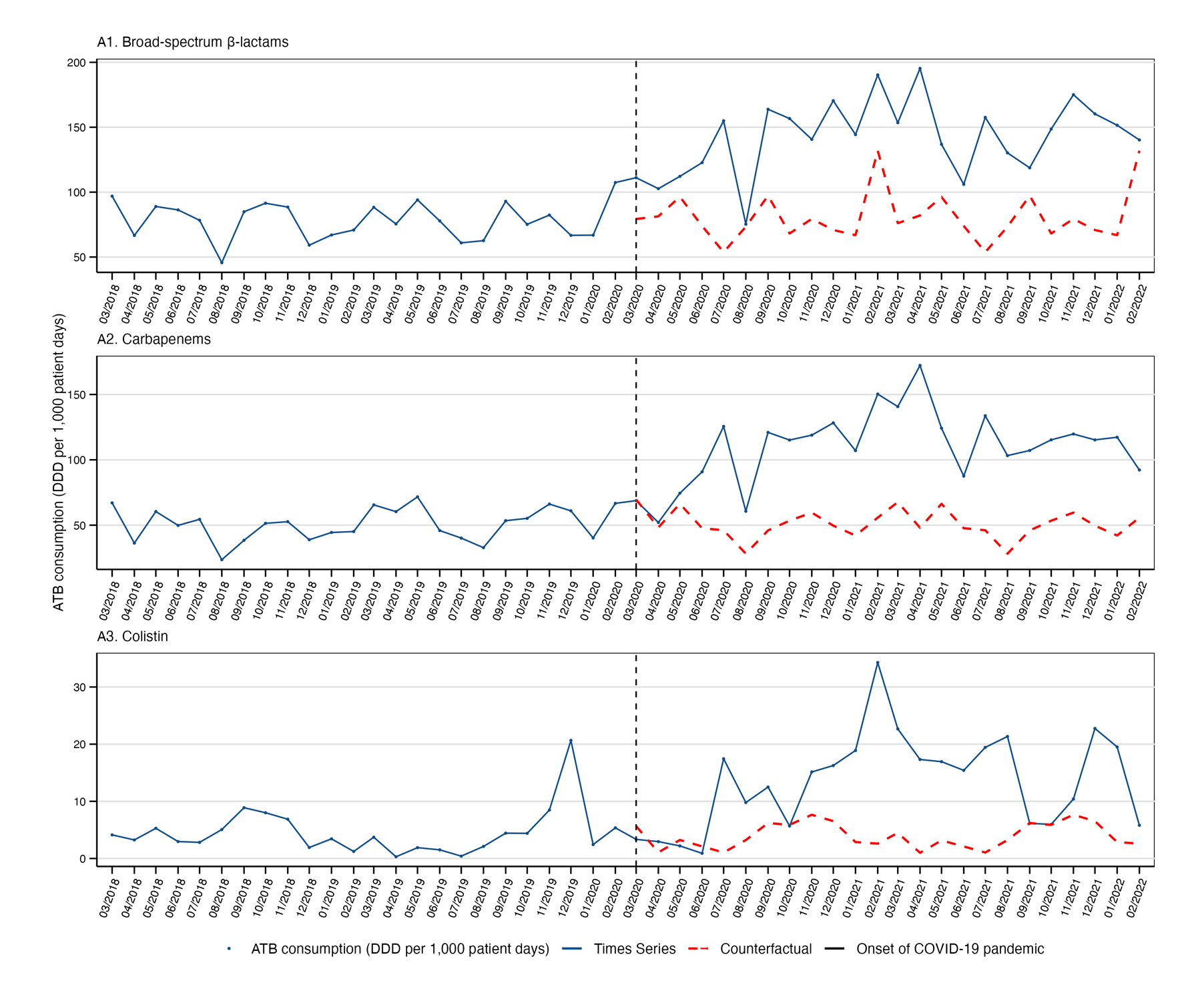


Notes: ATB= Antibiotic, Counterfactual scenario (dashed red lines) refer to a “COVID-19 free” scenario.

#
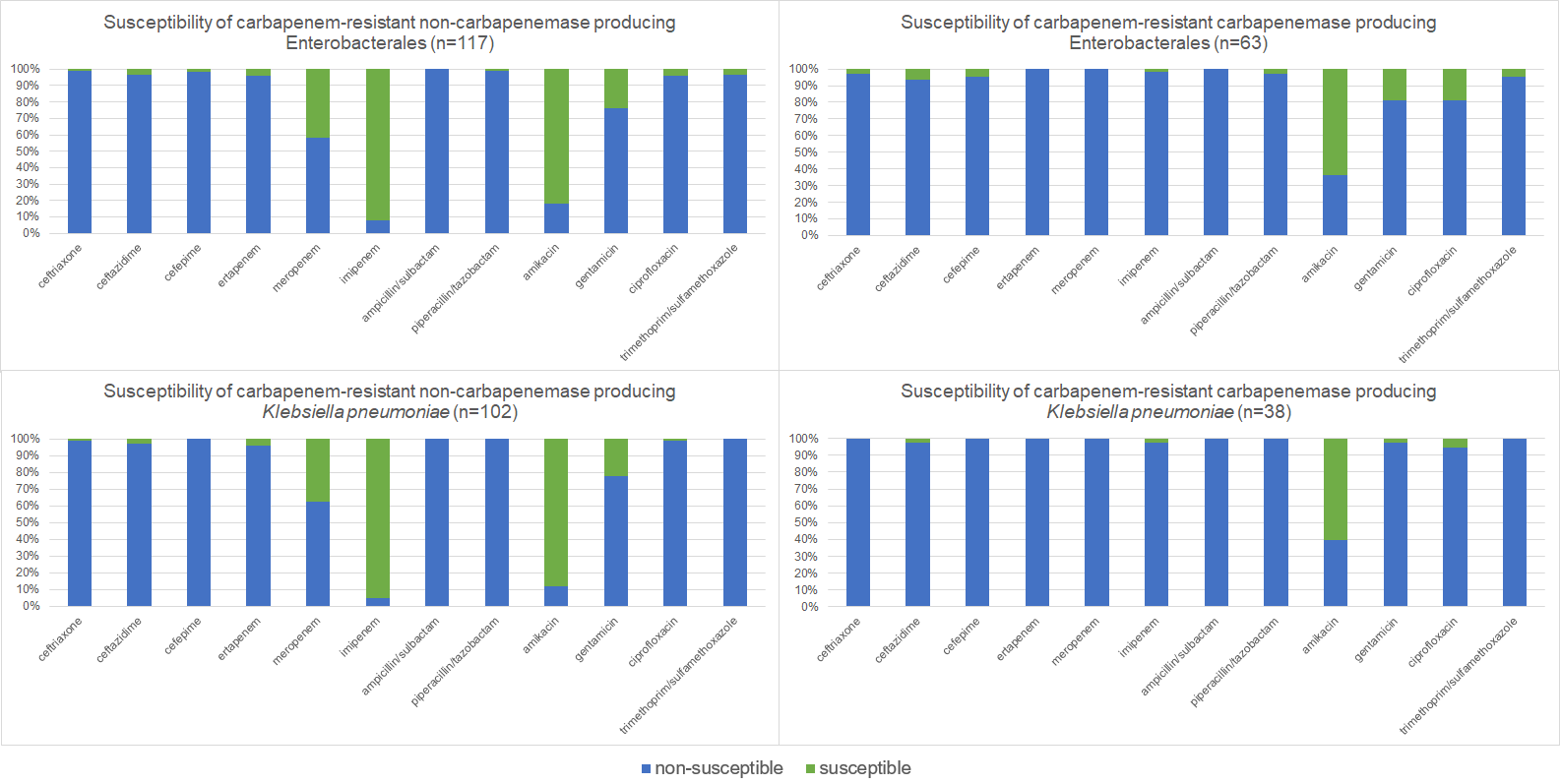
**Figure S6.** Susceptibility profile of carbapenemase-producing and non-producing carbapenem-resistant Enterobacterales and *Klebsiella pneumoniae* species from June 2018-February 2022

**Table S1.** Carbapenem-resistant Enterobacterales and proportion of carbapenemase-producing isolates harbouring *bla*_KPC_, *bla*_NDM_, or *bla*_VIM_ carbapenemases before and after COVID-19 pandemic onset, 2018–2022

|  | **Pre-COVID-19** | | **COVID-19** | | **Total** | |
| --- | --- | --- | --- | --- | --- | --- |
|  | n | % | n | % | n | % |
| **Total CRE** | 78 |  | 102 |  | 180 |  |
| PCR positive | 10 | 13% | 53 | 52% | 63 | 35% |
| *bla*_NDM_ | 4 | 40% | 39 | 74% | 43 | 68% |
| *bla*_KPC_ | 3 | 30% | 9 | 17% | 12 | 19% |
| *bla*_VIM_ | 3 | 30% | 5 | 9% | 8 | 13% |
| **Total CR-*Klebsiella pneumoniae*** | 62 |  | 78 |  | 140 |  |
| PCR positive | 2 | 3% | 36 | 46% | 38 | 27% |
| *bla*_NDM_ | 0 | 0% | 25 | 69% | 25 | 66% |
| *bla*_KPC_ | 2 | 100% | 9 | 25% | 11 | 29% |
| *bla*_VIM_ | 0 | 0% | 2 | 6% | 2 | 5% |
| **Total CR-*Enterobacter cloacae*** | 16 |  | 16 |  | 32 |  |
| PCR positive | 8 | 50% | 13 | 81% | 21 | 66% |
| *bla*_NDM_ | 4 | 50% | 11 | 85% | 15 | 71% |
| *bla*_KPC_ | 1 | 12% | 0 | 0% | 1 | 5% |
| *bla*_VIM_ | 3 | 38% | 2 | 15% | 5 | 24% |
| **Total CR-*Klebsiella ozaenae*** | 0 |  | 2 |  | 2 |  |
| PCR positive | 0 | - | 1 | 50% | 1 | 50% |
| *bla*_NDM_ | 0 | - | 0 | 0% | 0 | 0% |
| *bla*_KPC_ | 0 | - | 0 | 0% | 0 | 0% |
| *bla*_VIM_ | 0 | - | 1 | 100% | 1 | 100% |
| **Total CR-*Klebsiella variicola*** | **0** |  | **3** |  | **3** |  |
| PCR positive | 0 | - | 3 | 100% | 3 | 100% |
| *bla*_NDM_ | 0 | - | 3 | 100% | 3 | 100% |
| *bla*_KPC_ | 0 | - | 0 | 0% | 0 | 0% |
| *bla*_VIM_ | 0 | - | 0 | 0% | 0 | 0% |
| **Total CR-*Proteus mirabilis*** | **0** |  | **1** |  | **1** |  |
| PCR positive | 0 | - | 0 | 0% | 0 | 0% |
| *bla*_NDM_ | 0 | - | 0 | 0% | 0 | 0% |
| *bla*_KPC_ | 0 | - | 0 | 0% | 0 | 0% |
| *bla*_VIM_ | 0 | - | 0 | 0% | 0 | 0% |
| **Total CR-*Serratia marcescens*** | **0** |  | **2** |  | **2** |  |
| PCR positive | 0 | - | 0 | 0% | 0 | 0% |
| *bla*_NDM_ | 0 | - | 0 | 0% | 0 | 0% |
| *bla*_KPC_ | 0 | - | 0 | 0% | 0 | 0% |
| *bla*_VIM_ | 0 | - | 0 | 0% | 0 | 0% |

CRE, Carbapenem-resistant Enterobacterales; PCR, Polymerase Chain Reaction; *bla*_KPC_, *Klebsiella pneumoniae* Carbapenemase; *bla*_NDM_, New Delhi Metallo-β-lactamase; *bla*_VIM_, Verona Integron-encoded metallo-β-lactamase; CR, Carbapenem-resistant. Pre-COVID-19 period, March 2018 - February 2020; Post-COVID-19 period, March 2020 - February 2022.
